# Supplementary material for: MetaRibo-Seq measures translation in microbiomes
Source: Nat Commun. 2020 Jun 29;11:3268. doi: 10.1038/s41467-020-17081-z (PMC7324362; doi:10.1038/s41467-020-17081-z)
Supplement: Supplementary file 10 — Supplementary Data 7 [file 41467_2020_17081_MOESM10_ESM.zip › File2/Confidence_High_Taxonomy/121209_out.krona.html]

Javascript must be enabled to view this page.

members
magnitude
magnitudeUnassigned
count
unassigned
taxon
rank

121209\_out

3

superkingdom
3
2

1239
3
phylum

class
186801
3

3
186802
order

1
31979
family

genus
1485
1

species
1506
1

SRS077502\_contig\_number\_contig-100\_1297.114161

186803
2
family

1506577
2
genus

species
2
29361

SRS075398\_contig\_number\_719SRS142781\_contig\_number\_contig-100\_2489.45547
